# An Improved Neutral α-Glucosidase Assay for Assessment of Epididymal Function—Validation and Comparison to the WHO Method

**DOI:** 10.3390/diagnostics4010001

**Published:** 2014-01-09

**Authors:** Frank Eertmans, Veerle Bogaert, Tanita Van Poecke, Barbara Puype

**Affiliations:** 1 Department of Research and Development, FertiPro NV, Industriepark Noord 32, 8730 Beernem, WVL, Belgium; E-Mails: veerle@fertipro.com (V.B.); barbara@fertipro.com (B.P.); 2 Laboratory of Andrology, Ghent University Hospital, De Pintelaan 185, 9000 Ghent, OVL, Belgium; E-Mail: tanita.vanpoecke@uzgent.be

**Keywords:** epididymis, neutral α-glucosidase, method optimization, assay validation

## Abstract

Neutral α-glucosidase (NAG) activity in human seminal plasma is an important indicator for epididymis functionality. In the present study, the classic World Health Organization (WHO) method has been adapted to enhance assay robustness. Changes include modified enzyme reaction buffer composition and usage of an alternative enzyme inhibitor for background correction (glucose instead of castanospermine). Both methods have been tested in parallel on 144 semen samples, obtained from 94 patients/donors and 50 vasectomized men (negative control), respectively. Passing-Bablok regression analysis demonstrated equal assay performance. In terms of assay validation, analytical specificity, detection limit, measuring range, precision, and cut-off values have been calculated. These data confirm that the adapted method is a reliable, improved tool for NAG analysis in human semen.

## 1. Introduction

Semen α-glucosidase activity can be subdivided into a neutral (epididymis-related) and an acid (prostate-derived) fraction [1]. In patients with azoospermia and normal androgen levels, neutral α-glucosidase (NAG) activity in seminal plasma is a reliable parameter for epididymal function. Alternative markers, such as glycerophosphocholine and L-carnitine, are also available but NAG activity appears to be more specific and sensitive [2]. 

Seminal plasma of azoospermic males with bilateral obstruction between the epididymis and the ejaculatory duct contains very low α-glucosidase [3]. In contrast, enzyme activity is normal when azoospermia results from sperm maturation arrest, an obstruction located between the epididymis and the rete testis, or in the rete testis. Hence, NAG assessment in seminal plasma of normally virilized men with azoospermia allows differentiation between the major causes of this condition [4,5].

Low NAG in patients with oligozoospermia may reflect partial obstruction of the epididymides, associated with infections or inflammatory disease [3,6]. Enzyme activity in patients with normal sperm concentration is correlated with the outcome of the Shorr-stain of midpiece and tail, reflecting changes in the sperm membrane, induced by epididymal secretion [6].

A reliable method for NAG activity assessment in semen is described in the World Health Organization (WHO) manual for semen analysis [7]. Although being the gold standard, assay robustness can still be improved by changing various reaction parameters, including buffer composition and the use of an alternative inhibitor for background correction. A detailed overview of the applied changes is presented, together with a statistical comparison to the WHO method. Finally, validation parameters are discussed to verify assay performance and reliability. 

## 2. Experimental Section

### 2.1. Materials and Methods

All ingredients, including para-nitrophenol (PNP), para-nitrophenyl-α-D-glucopyranoside (PNPG), sodium dodecylsulfate (SDS), castanospermine, glucose, reaction buffer ingredients, and NaOH were purchased from Sigma Aldrich (Diegem, Belgium), Acros (Geel, Belgium) or Prolabo (Leuven, Belgium), respectively. The exact composition of the enzyme reaction buffer (pH 6.8) is considered proprietary and cannot be disclosed. 

The WHO method was performed as described in the WHO manual, using homemade reagents [7]. The improved NAG assay is commercially available (EpiScreen Plus™) and has been executed according to the instructions of the manufacturer (FertiPro NV, Beernem, Belgium). Assay principle and methodological changes to the WHO method are described in detail below. 

### 2.2. Assay Principle

Under specified conditions (pH = 6.8; T = 37 °C), α -glucosidase will catalyze the conversion of the substrate 4-nitrophenyl-α-D-glucopyranoside to α-D-glucopyranoside and p-nitrophenol, as shown below (Equation (1)). The yellow colour of the latter product is measured spectrophotometrically at 405 nm.


[PNPG + α-glucosidase] → α-D-glucopyranoside + PNP (yellow)]
(1)

As the reaction buffer contains SDS, the acid form of α-glucosidase (originating from the prostate), is selectively inhibited. This allows specific determination of neutral enzyme activity [1].

### 2.3. Enzyme Kinetics

Kinetic experiments have been performed according to the Michaelis-Menten model [8]. Briefly, linearity of substrate conversion, the Michaelis-Menten constant (Km), and maximal velocity (Vmax) have been assessed in high, medium and low activity semen samples (n = 13), incubated at pH 6.8 (37 °C). The following Equation (2) has been used:

[V = (Vmax × S) / (Km + S)]
(2)

With V = reaction velocity and S = substrate concentration. Experimental data were used to select optimal incubation period and the substrate concentration, yielding maximum reaction velocity.

### 2.4. Enzyme Activity Calculation

Alpha-glucosidase enzyme activity is expressed as mIU per mL or mIU per ejaculate (if corrected for ejaculate volume). At pH = 6.8 and T = 37 °C, one α-glucosidase unit is able to liberate 1 µmolar of PNP from PNPG per minute. Therefore, one has to take into account the semen dilution factor (improved assay: 1150/20 = 57.5; WHO: 1115/15 = 74.33) and the incubation time (2 h for both methods). This results in a correction factor of 0.479 and 0.619 for the improved and WHO test, respectively. The calculated PNP concentration for each sample is multiplied by this correction factor in order to convert to enzyme activity (mIU/mL).

### 2.5. Seminal Plasma Background

Glucose has been evaluated as an alternative for castanospermine. In total, 7 independent experiments have been performed using 9 different semen samples with variable activity. Dose response curves were generated by gradually increasing the concentration of castanospermine and glucose, respectively. Optical density (OD) was measured at 405 nm using a spectrophotometer (Zenyth 3100; Anthos Labtec, Heerhugowaard, The Netherlands). Values were expressed as a % of the OD value obtained for the non-inhibited sample (reference). 

### 2.6. Improved Protocol *vs.* WHO Method

All preliminary experiments contributed to the final assay protocol. In the next phase, the new assay protocol was extensively compared with the gold standard, being the WHO method [7]. This study was approved by the ethics committee of Ghent University Hospital (Ghent, Belgium). All patients have signed an informed consent, allowing semen samples to be used for research purposes. 

Briefly, 144 frozen seminal plasma samples of patients who visited the andrology laboratory of the University Hospital between 2009 and 2011 were randomly chosen. This selection was based on semen analysis results (α-glucosidase content) and clinical data, kindly provided by Ahmed Mahmoud (Laboratory of Andrology, Ghent University Hospital, Ghent, Belgium). They have been divided into a subset of 94 patient/approved donor samples (normal group) and a subset of 50 approved vasectomy samples (negative control), respectively. Samples analysis was performed in parallel, using both methods. Importantly, to allow reliable and unbiased method comparison, all tests were done in a single-blind manner by an independent laboratory technician of the Andrology laboratory.

### 2.7. Assay Validation

Assay validation has been performed according to the Clinical and Laboratory Standards Institute (CLSI) guidelines [9], including evaluation of analytical specificity, detection limit (sensitivity), measuring range, precision, and cut-off. 

### 2.8. Statistical Analysis

Beside descriptive statistics, other analyses, including Passing-Bablok regression, reference interval determination, linear regression (PNP standard curve), detection limit calculation, and receiver operating characteristic (ROC) curve analysis, have been performed using MedCalc statistical software (MedCalc Software BVBA, Ostend, Belgium). 

## 3. Results and Discussion

### 3.1. Assay Design

**Figure 1:**
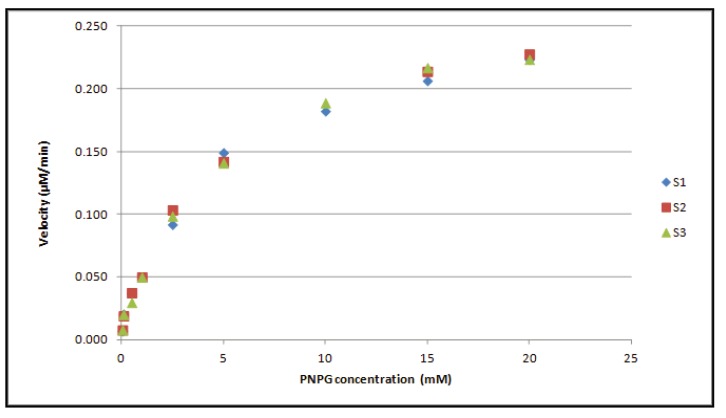
The Michaelis Menten curve represents the enzyme reaction velocity, expressed as µM/min, in function of substrate (PNPG) concentration. This figure is an example of the analysis of three different semen samples (S1–S3).

In our hands, addition of sodium dodecylsulfate (SDS) to the WHO phosphate buffer (pH 6.8) resulted in a strong, white precipitation. According to the monograph, precipitation will disappear by gentle warming. As we were not able to reproduce this setup, buffer composition has been modified to avoid precipitation formation. It is important to stress that no changes have been made to SDS content and pH, a crucial parameter for optimal enzyme activity. Enzyme kinetic properties (Michaelis Menten model) have been determined in order to evaluate the effect of the adapted reagent buffer on NAG activity. The correlation between substrate (PNPG) concentration and reaction velocity (µM/min) demonstrates that enzyme activity strongly depends on substrate concentration (Figure 1). 

The affinity between an enzyme and its substrate is reflected by the Michaelis-Menten constant (K_m_), being the substrate concentration at which 50% of maximum reaction velocity is achieved. In our hands, an average Km value of 4.604 ± 0.63 mM was calculated. The WHO method recommends a concentration of 16.6 mM to reach maximal reaction velocity. Although confirmed by our data, we decided to use a concentration of 20 mM in order to guarantee sufficiently high PNPG content, and thus optimal enzyme activity, during the complete shelf life of the commercial kit.

Regardless of sample enzyme activity, substrate conversion was linear between 90 and 210 min (PNPG concentration of 20 mM). However, the linear curve of some samples showed a small planatation if samples were incubated longer than 210 min (data not shown). Similar to the WHO method, we have chosen 120 min as final incubation period.

In summary, the selected assay conditions (adapted reagent buffer (pH 6.8) with SDS, PNPG concentration = 20 µM, 2 h incubation at 37 °C) will guarantee optimal enzyme activity, independent of the semen NAG content.

### 3.2. Enzyme Inhibition

**Figure 2:**
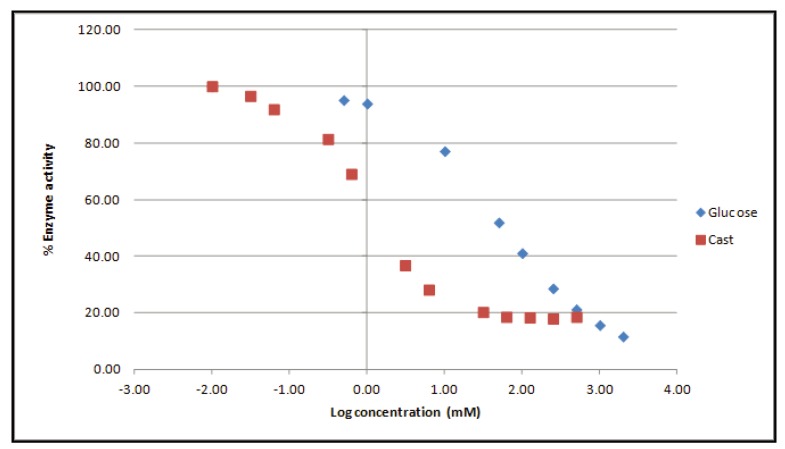
Both glucose (blue) and castanospermine (red) dose-dependently block NAG enzyme activity in seminal plasma. Castanospermine has a stronger inhibitory capacity in comparison to the carbohydrate.

Semen plasma is a complex mixture of biochemical compounds, originating from the prostate, the epididymis, the accessory glands, the ampullae and the seminal vesicles. It is important to stress that semen plasma composition strongly depends on individual gland secretion, thereby resulting in within- and between-patient variability [10]. In other words, correct enzyme activity calculation requires adjustment for seminal plasma background. For this purpose, the WHO method uses castanospermine, a strong inhibitor of α-glucosidase activity [7]. An interesting alternative is glucose, which blocks enzyme activity by competitive binding to the monosaccharide binding site of α-glucosidase [11]. This pH-dependent process is called competitive product inhibition. Indeed, inhibitory experiments demonstrate a dose-dependent reduction of NAG activity (Figure 2). In the improved protocol, the applied glucose concentration yields identical inhibition as the castanospermine reference.

The WHO advises using only two internal quality control samples for background correction. However, our experiments demonstrated that the background variance of semen samples is indeed quite large (>20%) for both glucose and castanospermine-treated samples (data not shown). Therefore, we recommend preparing a negative control for each seminal plasma sample in order to allow correct and reproducible background correction. Thus, NAG assay outcome will not be influenced by a variable semen plasma background.

### 3.3. Method Comparison

Both assays have been compared using the Passing and Bablok methods, a non-parametric regression analysis tool [12]. If both tests are identical, the linear regression formula yields a slope of 1 and an intercept of 0. The latter reflects systematic differences, while the slope represents proportional differences. If both 1 (slope) and 0 (intercept) are included in the respective 95% confidence intervals (CI), methods are comparable. Patient/donor (normal group) and vasectomy data were normally distributed (no outliers; data not shown). For both groups, no differences were found between the WHO method and the improved assay. When all samples (n = 144) were pooled, the 95% CI for the slope contained 1, while the zero value was not included into the 95% CI of the intercept. 

**Figure 3:**
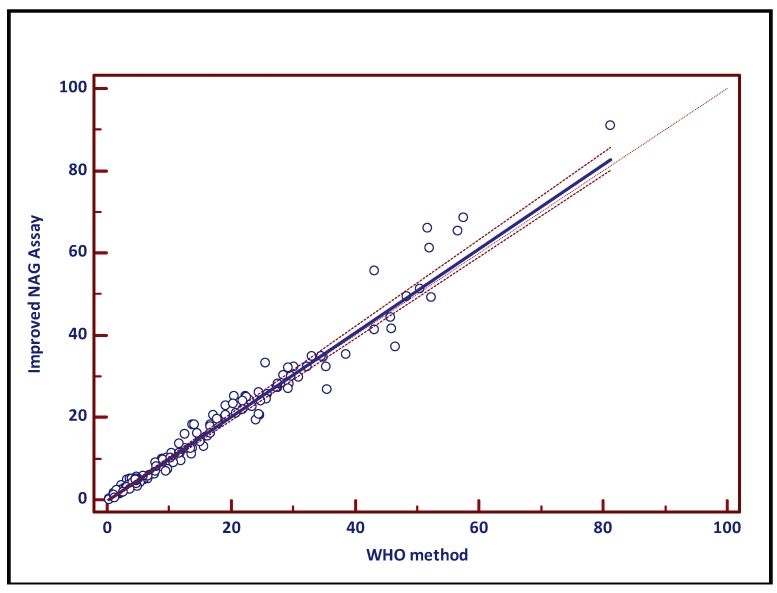
Passing-Bablok regression analysis demonstrates equal performance of both NAG methods (WHO, X-axis; improved NAG assay, Y-axis). Regression parameters are presented in Table 1.

Based on the experimental data, one may conclude that both methods vary by a constant amount (−0.3279). However, this value is marginally low and will not affect assay outcome. In other words, it is clinically irrelevant. This observation is confirmed by the results of the separate groups, showing no difference. Data are presented in Figure 3 and Table 1. 

**Table 1:** Passing-Bablok regression parameters.

Regression parameters	Normal	Vasectomy	Total
Slope	1.035581	0.9178	1.0228
Intercept	−0.622717	−0.106595	−0.3279
95% CI Slope	0.986 to 1.090	0.837 to 1.016	0.995 to 1.054
95% CI Intercept	−1.442 to 0.441	−0.285 to 0.103	−0.612 to −0.038

### 3.4. Assay Validation

#### 3.4.1. Analytical Specificity

As semen samples are complex biological samples, assessment of NAG activity must be robust and reproducible. Specificity of the improved protocol is guaranteed by: optimal reaction conditions, selective inhibition of acid α-glucosidase by SDS [1], the use of an enzyme-specific substrate (PNPG) at optimal concentration (maximal reaction velocity) [13], and background correction for each sample.

#### 3.4.2. Detection Limit

The detection limit is 2.32 and 2.11 mIU/mL for the improved and the WHO method, respectively. Both values have been calculated using Equation (3).


[Detection limit = (3.3 × SD) / S]
(3)

With SD = standard deviation of the analytical response, and S = slope of the PNP curve.

#### 3.4.3. Measuring Range of the Assay

For each assay run, a standard curve of PNP is included to determine PNP production and corresponding enzyme activity of test samples. The curve remains linear up to 300 µM (144 mIU/mL) but the assay curve ends at 200 µM (95.8 mIU/mL), as most samples do not exceed this value. This corresponds to a measuring range of 2.32–144 mIU/mL and 2.11–124 mIU/mL for the improved and the WHO method, respectively. The latter data confirm the sensitivity value, as stated by the WHO monograph (1.9 mIU/mL).

#### 3.4.4. Precision

Precision is defined as the ability of an assay to consistently reproduce a result when subsamples are taken from the same specimen. It is reflected by the intra-assay and interassay coefficient of variation (CV) [9]. These values have been calculated for two seminal plasma pools with low and enzyme high activity, using Equation (4).


[CV = 100 × (standard deviation / mean)]
(4)

The intra-assay CV was assessed by measuring 6× enzyme activity of both low and high pools, within the same run and performed by the same operator (lab technician). Interassay CV was calculated with data, obtained from 32 independent experiments, performed by multiple operators (3 lab technicians) at different days on both pools. Intra-assay CV values are 4.27/3.67% (low pool) and 1.88/1.47% (high pool) for the improved and the WHO method, respectively. Interassay CV values are 10.13/10.6% (low pool) and 10.91/13.92% (high pool) for the improved and WHO method, respectively. These data demonstrate that both assays yield comparable, low inter- and intra-CV values, especially in view of the complex sample. 

#### =3.4.5. Within-Laboratory Precision=

The CLSI guidelines now use the term within-laboratory precision to denote the total precision within the same facility using the same equipment [9,14]. Within-laboratory precision for the improved method is 0.96 mIU/mL (low pool) and 3.70 mIU/mL (high pool), respectively. 

#### 3.4.6. Cut-Off

Reference intervals have been calculated according to the CLSI C28-A3 guidelines [9]. Briefly, the upper limit of the right-sided 95% CI of the vasectomy (negative control; n = 50) group is calculated for both methods (*cf.* Table 2). 

**Table 2:** Right-sided reference intervals for the improved and WHO methods.

	Improved NAG protocol	WHO
Upper limit	6.35 mIU/mL	6.84 mIU/mL
	19.98 mIU/ejaculate	22.08 mIU/ejaculate
90% CI	5.09–7.37 mIU/mL	5.64–7.92 mIU/mL
	15.44–23.63 mIU/ejaculate	16.63–26.48 mIU/ejaculate

**Figure 4:**
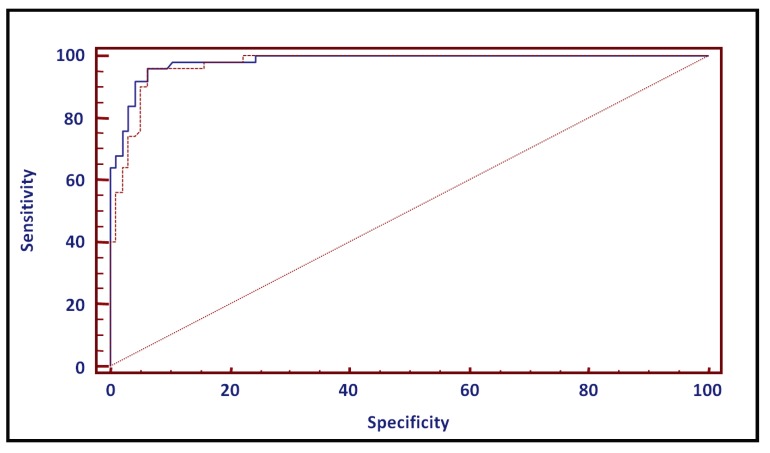
ROC Curve comparison (continuous line = improved assay, dotted line = WHO method).

A receiver operating characteristic (ROC) curve has been used as an alternative for cut-off calculation (Figure 4). The area under the curve (AUC) was 0.982 and 0.974 for the improved and WHO method, respectively. In other words, a randomly selected individual from the positive group has a test value larger than that for a randomly chosen individual from the negative group in 98.2 and 97.4% of the time. Both AUC values significantly differed from 0.5 (P < 0.0001), indicating that both methods are able to distinguish between the two semen sample groups. 

The highest specificity (93.6%) and sensitivity (96%) values are obtained for a cut-off value of 5.88 and 6.64 mIU/mL for the improved and WHO method, respectively. These data are almost identical to the values, obtained by the reference interval method. 

All described data are expressed in mIU/mL and are not corrected for ejaculate volume, which is advised by the WHO method. To evaluate the impact of ejaculate volume on assay outcome, both datasets have been multiplied with the respective ejaculate volume values and all statistical analyses were redone. All results were identical to the non-corrected data set, except for the ROC curves (data not shown). For both methods, specificity was almost identical (93.5 *vs*. 93.6%), but sensitivity was lower (93.6% *vs*. 96% for the improved assay and 92.6% *vs*. 96% for the WHO method). Cut-off values were 19.67 mIU/mL (improved assay) and 20.44 mIU/mL (WHO method). The latter value is identical to the cut-off value of the WHO monograph. 

## 4. Conclusions

Neutral α-glucosidase is a sensitive marker for detection of epididymis-related problems [2]. For this purpose, the WHO manual for semen analysis describes a reliable method for assessing NAG activity in human seminal plasma [7]. However, based on our experience, the robustness of the assay could be improved. First, the reaction buffer composition has been changed because, in our hands, the addition of SDS to the WHO phosphate buffer resulted in an irreversible precipitation. Sodium dodecylsulfate is required for inhibition of the prostate-derived, acid fraction, without affecting neutral activity [1]. The observed precipitation can be explained by the presence of potassium cations in the WHO phosphate buffer, which are produced by dissociation of potassium phosphate salts during dissolution. These cations will form a complex with dodecylsulfate anions (derived from SDS), which has a much lower solubility than its sodium salt variant. To prevent precipitation during buffer production, ingredients have been changed without affecting pH. Based on literature data, optimal enzyme activity requires a pH of 6.8 at 37 °C [13]. 

Enzyme kinetic studies have demonstrated that changing enzyme buffer composition has no impact on α-glucosidase activity. The average K_m_ (of 4.604 ± 0.63 mM) was comparable to the observations of Chapdelaine and colleagues (2.92 ± 0.84 mM) [13]. Substrate concentration in the improved assay is somewhat higher when compared to the WHO method (20 *vs.* 16.6 mM). Although both concentrations will result in maximum reaction velocity, the use of 20 mM PNPG guarantees optimal enzyme activity during the shelf life of the commercial kit. Finally, an incubation period of 2 h, which is identical to the WHO method, allows linear conversion of the PNPG substrate independent of sample activity. 

The second major change is the use of glucose instead of castanospermine for seminal plasma background determination. Although the former has stronger inhibitory properties, glucose concentration in the improved assay elicits identical enzyme inhibition as the castanospermine reference. Glucose blocks α-glucosidase activity by competitive binding to the monosaccharide binding site of α-glucosidase, a pH-dependent process called competitive product inhibition [11]. Replacing castanospermine by glucose not only reduces assay cost but more importantly, the significant increase in stability allows longer shelf life. Although the WHO method states to use only two quality control samples for background determination, we advise to analyze all samples because of the relatively high deviation among samples. This must allow a more accurate determination of NAG activity in semen. 

An extensive comparison to the golden standard (WHO) method has demonstrated identical analytical performance, as reflected by regression analysis and validation data. However, some remarks must be made. Passing-Bablok analysis revealed that both methods differ by a constant amount. However, the difference is marginally low and has no impact on assay outcome. Another important issue is the decrease of assay sensitivity if data were corrected for ejaculate volume, as observed with both methods. 

In conclusion, although the WHO method is an excellent and reliable tool for NAG analysis in human seminal plasma, assay robustness has been further improved by changing the reaction buffer composition and the type of inhibitor for background determination, without affecting assay performance.
